# Developing a method of implementation of screening, brief intervention, and referral to treatment in primary health care settings of Brazil and Latin America

**DOI:** 10.1186/1940-0640-8-S1-A6

**Published:** 2013-09-04

**Authors:** Pedro H Antunes da Costa, Daniela Cristina Belchior Mota, Erica Cruvinel, Fernando Santana de Paiva, Telmo Mota Ronzani

**Affiliations:** 1 Federal University of Juiz de Fora, Brazil; Center for Research, Intervention and Evaluation for Alcohol & Drugs (CREPEIA

## 

Screening, brief intervention, and referral to treatment (SBIRT) appears to be an important tool in Brazil and Latin America to address the use/abuse of alcohol and other drugs and to help implement and organize preventive activities, particularly in the services of primary health care (PHC). Nevertheless, there are few studies evaluating the implementation of SBIRT in PHC of Latin American countries, identifying elements that promote positive results in the prevention and/or reduction of consumption according to each situation. Therefore, this study presents a method for implementing SBIRT developed in the PHC of a small/medium Brazilian city, highlighting challenges and possibilities for its dissemination in other cities and countries in Latin America. This is a research intervention, with data collection and evaluation through participant observation. The entire process was evaluated to the suitability to local needs, determining factors that could facilitate or impede its implementation. The method developed consisted of six stages: initial contact and planning, diagnosis and mapping, raising awareness, training and monitoring. In the process of implementation the hindering points detected were: insufficient resources (human, financial, infrastructure), shortage of integration and intersectional health care settings, lack of participation by physicians, focus in dependence, scarcity of participation of civil society and absence of spaces where this population could participate. However, the participation of community health workers and nurses in the implementation and organization of SBIRT, the actions implemented by health teams, such as educational and preventive practices in schools and discussion groups with communities, are positive prospects for the implementation of SBIRT in the Latin American PHC context. Finally, the method presented is considered a useful model to other Latin American cities and countries, with necessary adaptations. Despite the advances, progress is needed in preventing alcohol and other drug abuse in Latin America, with long-term measures.

